# Efficacy and radiographic analysis of oblique lumbar interbody fusion for degenerative lumbar spondylolisthesis

**DOI:** 10.1186/s13018-019-1416-2

**Published:** 2019-11-28

**Authors:** Menghui Wu, Jia Li, Mengxin Zhang, Xufeng Ding, Dongxu Qi, Guimiao Li, Yong Shen

**Affiliations:** Department of Orthopedic Surgery, Hebei Medical University Third Affiliated Hospital, No. 139 Ziqiang Road, Shijiazhuang, 050051 China

**Keywords:** Lumbar spondylolisthesis, Posterior lumbar interbody fusion, Oblique lumbar interbody fusion efficacy, Radiographic

## Abstract

**Background:**

To compare the clinical efficacy and radiographic analysis of oblique lumbar interbody fusion (OLIF) and traditional posterior lumbar interbody fusion (PLIF) in treating degenerative lumbar spondylolisthesis (DLS).

**Methods:**

Grade I DLS patients admitted to the Third Hospital of Hebei Medical University were retrospectively reviewed. In sum, 78 patients that underwent OLIF (*n* = 31) and PLIF (*n* = 47) treatment of DLS were recruited. Clinical data including clinical and radiological evaluations were collected pre-operatively and at each follow-up. Japanese Orthopaedic Association (JOA) scores, Oswestry Disability Index (ODI), lumbar lordosis (LL), disc height (DH), and fusion rates were compared between the OLIF and PLIF groups.

**Results:**

The operation time for both groups was 131.3 ± 14.6 min in the OLIF group and 156.9 ± 37.4 min in the PLIF group (*P* < 0.001). The intraoperative blood loss was 163.6 ± 63.9 ml in the OLIF group and 496.8 ± 122.6 ml in the PLIF group (*P* < 0.001). The length of the surgical incision was 4.63 ± 0.57 cm in the OLIF group and 11.83 ± 1.37 cm in the PLIF group (*P* < 0.001). The number of intraoperative and post-operative complications for both groups was 10 in the OLIF group and 20 in the PLIF group. Significant clinical improvement (*P* < 0.05) was observed in JOA scores and ODI when comparing pre-operative evaluation and final follow-up. After statistical analysis, there was no significant difference in the preoperative JOA scores between the two groups. There was no significant difference when comparing pre-operative LL and DH for either group. Post-operative reexamination was improved as compared to pre-operative exams. And the improvement of DH was better in the OLIF group as compared to the PLIF group.

**Conclusions:**

For DLS patients, both OLIF and PLIF can achieve good results. Furthermore, OLIF displays marked advantages including smaller surgical incisions, shorter anesthesia times, decreased intraoperative blood loss, and post-operative pain better relieved.

## Introduction

Lumbar spondylolisthesis (LS) is a common chronic disease of the human population, with an incidence of about 6% [1]. LS is essentially a slip of the upper vertebral body relative to the lower vertebral body. Accordingly, the cause of LS can be divided into degenerative, isthmic fissure, dysplasia, traumatic, and pathological, among which degenerative lumbar spondylolisthesis (DLS) is the most common. Treatment consists of conservative and surgical approaches.

The surgical plan typically includes posterior lumbar fusion, lumbar posterolateral fusion, posterior lumbar interbody fusion (PLIF), oblique lateral interbody fusion (OLIF) [2], and other additional surgical interventions. OLIF represents a new surgery technique that was originally introduced by Mayer [3] in 1997, which is a minimally invasive oblique lateral retroperitoneal technique, which was further improved by Silvestre [2].

In recent years, OLIF has been widely promoted, and PLIF, which is represented by a traditional surgical plan, is the currently accepted surgical procedure in treating DLS, and the curative effect is well established and confirmed. In recent years, OLIF is a recommended surgical procedure exhibiting obvious advantages over PLIF. In addition, lumbar sagittal radiographic parameters and disc height (DH) are important in the occurrence and development of DLS [4–7] and should be used as a therapeutic evaluative standard.

In view of the currently reported high incidence of DLS, and the need for important treatment of affected patients, this article will explore the advantages of OLIF in treating DLS and will do so by comparing the post-operative outcomes of both procedures.

## Materials and methods

### Inclusion criteria

In patients with DLS grade I, neurological signs were consistent with the presenting symptoms and radiological outcomes confirmed lumbar instability. All patients were treated with regular conservative therapy for more than 3 months; however, treatment was ineffective or the symptoms were aggravated. The follow-up time ranged from 14 to 28 months. Among them were 9 males and 22 females in the OLIF group, with a mean age of 60.0 years (range 43–78 years), and 17 males and 30 females in the PLIF group, with a mean age of 57.3 years (range 44–71 years).

### Exclusion criteria

Patients were excluded if they presented with grade II DLS and above, traumatic injury, multi-segment lumbar disc herniation, congenital spinal deformity, severe lumbar spinal stenosis, intervertebral space infection, or spinal tumors.

### OLIF surgery

**Fig. 1:**
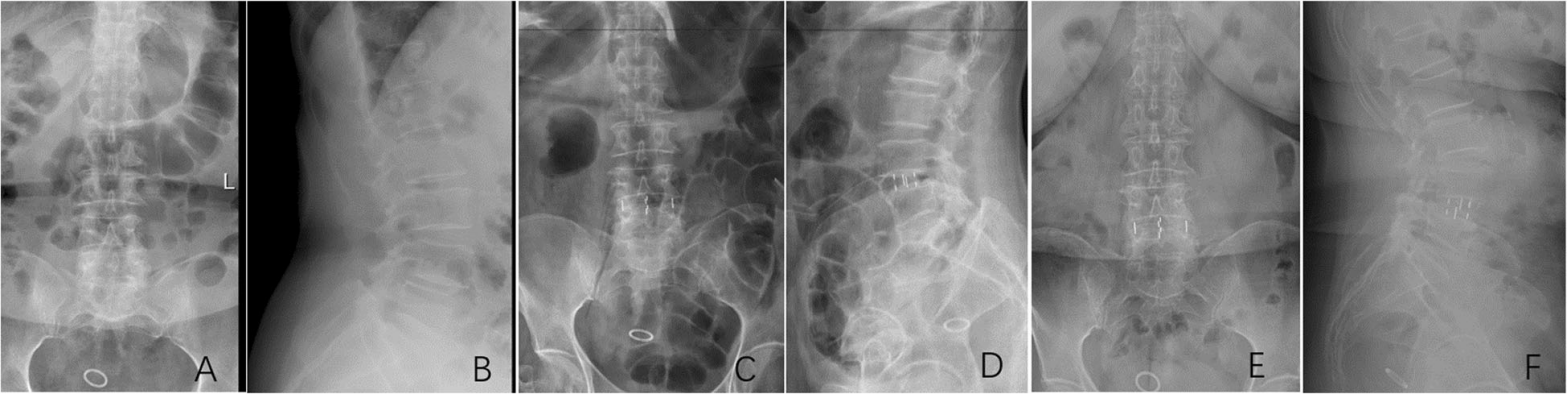
A 63-year-old woman suffered from low back and bilateral lower limb pain for 6 years, aggravating for 4 months. X-ray of the lumbar spine showed a typical case of single-level OLIF for L4 degenerative spondylolisthesis. **a** Preoperative anteroposterior radiograph. **b** Preoperative lateral radiograph. **c** Postoperative anteroposterior radiograph. **d** Postoperative lateral radiograph. **e** The last follow-up anteroposterior radiograph. **f** The last follow-up lateral radiograph

In this procedure, a small incision of about 4.5 cm on the front line of the iliac crest was made at the left lower abdomen and blunt forceps were used to separate subcutaneous tissue, obliquus externus abdominis, obliquus internus abdominis muscle, and transverse abdominis muscle. The lumbar vertebral body was revealed through the backside of the peritoneum and was located between the front psoas muscle and the large vascular. After X-ray positioning, the annulus fibrosus was resected and placed into a dedicated progressive dilator and the working channel. This procedure also required scraping of the intervertebral disc tissue with a specific instrument. During the process, it was important to avoid damage to the bony endplate, following which a proper sized cage was inserted, and then, the intervertebral space was opened, while confirming the position and size by X-ray fluoroscopy, and then, the surgical incision was sutured layer by layer. (Fig. 1).

### PLIF surgery

**Fig. 2:**
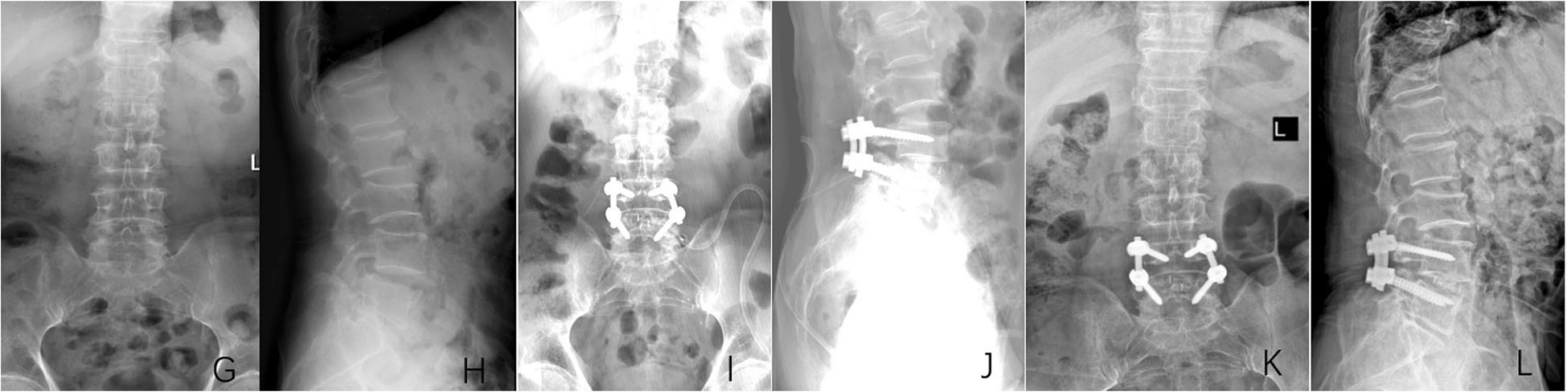
A 69-year-old male patient suffered from low back and left lower limb pain for 2 years, aggravating for 1 month. X-ray of the lumbar spine showed a typical case of single-level PLIF for L4 degenerative spondylolisthesis. **g** Preoperative anteroposterior radiograph. **h** Preoperative lateral radiograph. **i** Postoperative anteroposterior radiograph. **j** Postoperative lateral radiograph. **k** The last follow-up anteroposterior radiograph. **l** The last follow-up lateral radiograph

This procedure is accomplished by making an incision in the middle of the back of the waist, wherein the scalpel makes an incision to the skin, the subcutaneous tissue, and the fascia, via a layer-by-layer approach. Electrosurgical separation of the paraspinal tissue and paravertebral tissue under the periosteum was completed, following which the pedicle screw was positioned into the pedicle of the vertebral arch. Pedicle screw was used in all PLIF patients. The upper rod was used to open the intervertebral space and restore the vertebral body by moving forward to the original position. The crypts on both sides were fully decompressed, the ligamentum flavum and bone tissue situated close to the outlet root were completely cleaned, and the safety triangle was used to treat the intervertebral disc. After implanting the bone block, a proper sized cage was placed, and the incision was washed using normal saline, following which the drainage tube was placed in the surgical incision and the incision was sutured layer by layer. (Fig. 2).

### Follow-up and evaluation indicators

According to the pre-operative, the post-operative, and the final follow-up of the JOA scores, we could evaluate improvements of lower back pain in the pre- and post-operative stages of both groups of patients. We used the pre-operative, post-operative, and the final follow-up of the ODI score to assess improved lumbar spine function. According to the post-operative X-ray outcome, which determined the degree of interbody fusion, we could evaluate improved DH and LL.

### Statistical processing

All statistical analyses were conducted using the SPSS version 21.0 statistical software program. The count data was expressed as a percentage, and the measurement data was expressed as mean ± SD. The comparisons between pre- and post-operative parameters within groups were performed by the paired Student’s *t* test, and independent sample Student’s *t* test was used to compare between groups. Chi-square test was used to analyze categorical variables. An alpha value of *P* < 0.05 was considered statistically significant.

## Results

There were no significant differences when comparing the OLIF and the PLIF group for all base-line patient characteristics that were assessed (Table 1). The post-operative JOA scores for both groups were significantly improved (*P* < 0.001). There was also a statistically significant difference in JOA scores when comparing both groups (*P* < 0.001). The reported improvement in the lower back pain for the OLIF group was a better improvement when compared to the PLIF group. At the final follow-up, there were further improvements for both groups (*P* < 0.001). No significant differences were seen in terms of the final follow-up when comparing both groups (*P* = 0.09). That is, both groups had similar recovery at the final follow-up. The pre-operative ODI scores for both groups were similar (*P* = 0.713). The post-operative ODI scores of both groups were significantly improved as compared to the pre-operative ODI scores (*P* < 0.001). In addition, there was a statistically significant difference in ODI scores when comparing both groups (*P* = 0.02). An improvement in lumbar function for the OLIF group was better than that seen for the PLIF group. Functional recovery was also superior in patients of the PLIF group. At the final follow-up, lumbar function was further restored (*P* < 0.001). There was also no significant difference found when comparing both groups at the final follow-up (*P* = 0.258). The JOA and ODI scores for both groups were significantly improved as compared to those at pre-surgery (*P* < 0.001); however, the post-operative JOA and ODI scores in the OLIF group were more noticeably improved as compared to the PLIF group. OLIF demonstrated a better improved curative effect in the early post-operative period (Table 2).

**Table 1:** Baseline characteristics

	OLIF	PLIF	*P* value
Follow-up, months	18.00 ± 2.87	18.87 ± 3.47	0.255
Average age, years	60.0 ± 9.3	57.3 ± 7.0	0.235
Gender			0.513
Male	9	17	
Female	22	30	
Fused segments			0.676
Single level	26	41	
Double level	5	6

**Table 2:** Preoperative, postoperative, and final follow-up’ JOA scores and ODI scores of the two groups of patients

	JOA scores		ODI	
	OLIF	PLIF	OLIF	PLIF
Preoperative	13.23 ± 1.16	13.70 ± 1.35	59.7% ± 6.3%	59.1% ± 6.8%
Postoperative	22.67 ± 1.33*	20.34 ± 1.26*	25.1% ± 4.9%*	28.5% ± 7.7%*
Final follow-up	24.83 ± 1.53	23.58 ± 2.01	14.8% ± 6.3%	16.7% ± 6.8%

*ODI* Oswestry Disability Index, *JOA* Japanese Orthopaedic Association

*Statistically significant versus preoperative values (*p* < 0.05)

There was a statistically significant difference in the surgical procedure time when comparing both groups (*P* < 0.001). A significant difference was found for the amount of intraoperative blood loss for both groups (*P* < 0.001). Additionally, the surgical incision length when comparing both groups was significantly different (*P* < 0.001; Table 3).

**Table 3:** Comparison of intraoperative and situation between the two groups of patients

	OLIF group	PLIF group	*P* value
Operation time	131.3 min ± 14.6 min	156.9 min ± 37.4 min	*P* < 0.001
Amount of bleeding	163.6 ml ± 63.9 ml	496.8 ml ± 122.6 ml	*P* < 0.001
Incision length	4.63 cm ± 0.57 cm	11.83 cm ± 1.37 cm	*P* < 0.001

In terms of post-operative complications, we found the following in the OLIF group: three cases of transient thigh pain and/or numbness, two cases of transient thigh flexion weakness, one case of dural tear, one case of peritoneal injury, and three cases of cage sinking for a total of ten cases. In the PLIF group, we found the following: one cases of transient thigh pain and/or numbness, one case of transient thigh flexion weakness, six cases of dural tear, two cases of post-operative epidural hematoma, five cases of post-operative incision infection or poor healing, one case of cage sinking, and four cases that developed intermuscular venous thrombosis for a total of 20 cases. Intermuscular venous thrombosis easily occurred in the PLIF group because of more nerve root stimulation during the operation, later movement, and less activity after the operation. The sample size of this group of patients is relatively small, and there may be some problems such as large systematic errors, resulting in intermuscular venous thrombosis in PLIF group is quite high than the other existing researches. In terms of intra- and post-operative complications, OLIF exhibited certain advantages in the dural tear, incision infection, poor healing, and intermuscular venous thrombosis. Of course, in terms of transient thigh pain and/or numbness and transient thigh flexion weakness and cage sinking, we determined that OLIF was somewhat inadequate and the incidence was higher than that found in PLIF (Table 4). This is a shortcoming of my writing. I have indicated in the table which complications are lower than those in PLIF group, and each of them has been tested by the chi-square test.

**Table 4:** Comparison of complications between the two groups

	OLIF group	PLIF group	*P* value
Transient thigh pain and/or numbness	3	1	0.014
Transient thigh flexion weakness	2	1	0.034
Dural tear	1	6	0.04
Postoperative epidural hematoma	0	2	0.094
Incision infection, poor healing	0	5	0.013
Peritoneal injury	1	0	0.215
Cage sinking	3	1	0.014
Intermuscular venous thrombosis	0	4	0.023

Bone fusion was obtained at the final follow-up of both groups, and there was no difference in fusion rate. The pre-operative LL of both groups were similar (*P* = 0.929). The post-operative LL of both groups was also improved, and a statistical difference was found when comparing both groups, including the OLIF (*P* < 0.001) and PLIF group (*P* < 0.001). There was no significant difference in post-operative LL found between both groups (*P* = 0.778), that is, both surgical procedures could be improved, although not to a level of statistical significance.

At final follow-up, although the LL of both groups had decreased slightly, the data were not statistically significant, including that found for OLIF (*P* = 0.915) and PLIF patients (*P* = 0.523). Furthermore, there was no significant difference when comparing both groups for post-operative LL (*P* = 0.682), and they were thus quite similar in LL at the final follow-up. Pre-operative disc surgery was also similar (*P* = 0.499). The post-operative DH infection that was determined for both groups was significantly higher than was found at the pre-operative stage (*P* < 0.001). There was also a statistically significant difference found for any improvement in the post-operative DH between both groups (*P* < 0.001), which indicated that an improvement in DH for the OLIF group was better than that found in the PLIF group. At the final follow-up, although DH was lost to varying degrees, the data were not statistically significant, and this observation included both OLIF (*P* = 0.054) and PLIF patients (*P* = 0.086). Data were also statistically significant (*P* < 0.001) at the final follow-up for both groups. Thus, the improvement found in the OLIF group was superior to that of the PLIF group (Tables 5 and 6).

**Table 5:** Improvement of preoperative, postoperative, and the final follow-up’ lumbar lordosis in two groups of patients

	Preoperative	Postoperative	Final follow-up
OLIF group	43.1° ± 12.1°	51.4° ± 10.6°*	50.4° ± 9.4°
PLIF group	43.3° ± 11.3°	49.7° ± 10.4°*	49.4° ± 10.0°

*Statistically significant versus preoperative values (*P* < 0.05)

**Table 6:** Improvement of preoperative, postoperative, and the final follow-up’ disc height in two groups of patients

	Preoperative	Postoperative	Final follow-up
OLIF group	8.06 mm ± 1.68 mm	12.67 mm ± 1.09 mm*	12.59 mm ± 1.11 mm
PLIF group	8.24 mm ± 1.31 mm	11.20 mm ± 1.02 mm*	11.13 mm ± 1.28 mm

*Statistically significant versus preoperative values (*P* < 0.05)

## Discussion

DLS is considered a degenerative disease of the aged and exhibits an increasing incidence in today’s society across the globe. It is apparent that at this time, the incidence of DLS is progressively increasing and the discomfort caused by intractable lower back pain, numbness, weakness, and other discomforts that are caused by lower limb nerve compression seriously affects the quality of life of affected patients [8, 9]. DLS also displays a certain degenerative imbalance and thus presents a risk factor for degenerative scoliosis in later life. This is in large part due to conservative treatment options being relatively inferior, thus indicating that surgical treatments offer a more meaningful approach [10].

### Surgical difference

OLIF is a relatively respected practice that has gained attention in more recent years. The surgical plan is an indirect decompression procedure [11–13]. During the operation, the intervertebral cage is used to expand the degenerative segment and stretch the soft tissue such as the ligamentum flavum in the spinal canal, thereby expanding the volume of the spinal canal and the intervertebral foramen with the intention of relieving neurological symptoms and lower back pain [14–16]. In addition, the surgical approach does not require separation of the paravertebral tissue and does not require surgical approaches that might include removal of the bone mass near the nerve root canal, for example, joint protrusion. This operation displays decreased nerve stimulation and avoids post-operative lower limb pain and numbness that is caused by intraoperative nerve stimulation.

By contrast, PLIF imparts low degrees of damage to normal tissues of the lumbar spine and paravertebral and no effect on the integrity of the three-column structure of the spine. PLIF is thus a direct decompression [17]. Due to the surgical field of vision and decompression requirements, relatively large surgical incisions and extensive separation of the paravertebral tissue are required to relieve the articular joints of the upper and lower vertebral bodies of the degenerative segment. To better perform nerve decompression, it is necessary to remove the inferior articular process of both sides of the upper vertebral body to ensure that there is sufficient decompression of the walking roots. These procedures can lead to severe damage of the lumbar vertebral bodies and paravertebral structures. Moreover, these destructive actions might also provoke post-operative refractory lumbosacral pain and other complications [18]. The separation of the paravertebral tissue and the larger incision is also a key reason that accounts for the high level of intraoperative blood loss and high incidence of post-operative incision complications. Altogether, both OLIF and PLIF display obvious differences in terms of the surgical procedure, and it presents as a key reason affecting post-operative efficacy.

### Comparison of clinical result: OLIF vs PLIF

The OLIF surgical plan is an indirect decompression method. The osseous structure of the spine was not destroyed during the operation. According to the pre-operative, post-operative, and the final follow-up assessment of lower back pain JOA and ODI scores [19], it can be concluded that the patient can obtain satisfactory surgical results in both the short and long terms post-surgery.

PLIF is a classic and effective surgical treatment for DLS and can be used in all types of DLS patient. The PLIF surgical method fully removes the soft tissue and bony structures of the nerve root canal and does so to ensure the thoroughness of nerve root decompression. In addition, the internal fixation system of the nail bar after the reduction procedure also guarantees its stability and long-term curative effect. However, due to the large peeling damage of the paravertebral tissue during the operation, the stability of the posterior column structure in the three-column structure of the spine was destroyed [20]. In addition, the incidence of intraoperative-related complications in PLIF was higher than that found for OLIF and the short-term effectiveness of PLIS was slightly insufficient as compared to OLIF. In particular, post-operative symptoms might be aggravated in the short term, or symptoms might not be relieved. Further, subjective feelings of the patients in the context of PLIS are found to be quite poor following surgery. Of course, OLIF also has the risk of sinking the cage, post-operative numbness of the waist, and leg pain [21–23], as well as peritoneal injury, ankle vascular injury, urinary system injury, and dural sac tear [24, 25].

In this current study, three DLS patients had transient thigh pain and/or numbness and two cases of transient thigh flexion weakness. After treatment with nerve swelling, the symptoms disappeared. Dural tear was found in one case, and after treatment with adequate fluid replacement, the patient had no obvious discomfort. Further, peritoneal injury was found in one case, with no obvious discomfort after surgery, and three cases of cage sinking at follow-up were found, although clinical symptoms were absent in this situation. Finally, bone fusion was found at the final follow-up. Intermuscular venous thrombosis was easily found in the PLIF group because of increased nerve root stimulation during surgery, in addition to latent movement and decreased activity following the procedure. In general, compared with the PLIF surgical plan, many of the complications from OLIF are mild, and the incidence of those complications is indeed low, wherein serious complications are quite rare. Subjective perceptions experienced by patients and objective data that are related to surgery have shown that OLIF exhibits several clear advantages.

### Radiographic analysis

The study found that the loss of lumbar curvature and changes in DH were closely related to the occurrence and progression of DLS. Therefore, it is of great clinical significance to study changes in LL and DH in patients with DLS. Loss of LL [26] is a key cause of lower back pain in patients and can be found by pre- and post-operative X-ray plain LL. Moreover, irrespective of whether it is OLIF or PLIF, LL can be recovered significantly. Recovery of DH in the lesion segment significantly improved the compression of the corresponding segmental nerve root. Both surgical procedures can increase DH of the diseased segment. By contrast, OLIF is superior to PLIF in this regard, indicating that the OLIF procedure has higher value and an improved curative effect of localized conditions.

### Limitations

Of course, this study also suffers from some shortcomings. OLIF is a relatively new operative procedure, and thus, the follow-up time of cases is slightly insufficient. It is not possible to make further comparative analyses of the long-term efficacy of both surgical methods. The intention is to follow up this study in the future to obtain further information aimed at improving the deficiencies identified in this article. In addition, there are some inadequacies in the number of cases, and the intention is to increase the number of follow-up cases in an attempt to reduce the error of follow-up data and to further improve the accuracy of this study. Of course, there are also other identified problems; for example, some patients did not undergo lumbar magnetic resonance examination during post-operative review and some patients conducted telephone follow-up, in the absence of further physical examination.

## Conclusions

Both OLIF and PLIF can achieve satisfactory clinical results in the treatment of DLS. The lumbar curvature and DH can also be improved significantly. However, compared with PLIF, OLIF requires a smaller surgical incision, exhibits a shorter anesthesia time, has decreased intraoperative blood loss, has decreased local tissue damage, and has earlier post-operative activities. In terms of any adverse clinical complications, in at least certain important aspects, OLIF has clear advantages as compared to PLIF.

## Data Availability

Data requests are available from the corresponding author.

## Abbreviations

DH: Disc height
JOA: Japanese Orthopaedic Association
LL: Lumbar lordosis
ODI: Oswestry Disability Index
OLIF: Oblique lumbar interbody fusion
PLIF: Posterior lumbar interbody fusion
